# Interleukin‐37 in Cancer Angiogenesis: Mechanisms, Therapeutic Potential, and Future Perspectives

**DOI:** 10.1002/cnr2.70580

**Published:** 2026-05-19

**Authors:** Elahe Saberi Teimourian, Mohammad Ali Karimi, Edessa Negera, Ghazaleh Behrouzian Fard, Anas Sattar, Mahdi Hosseini Bafghi, Nirusha Weerasinghe

**Affiliations:** ^1^ School of Health, Sports and Biosciences University of East London London UK; ^2^ Department of Laboratory Sciences, Faculty of Paramedical and Rehabilitation Sciences Mashhad University of Medical Sciences Mashhad Iran

**Keywords:** angiogenesis, cytokine‐based therapy, Interleukin‐37, tumor immunology, tumor microenvironment

## Abstract

**Background:**

Interleukin‐37 (IL‐37), a member of the interleukin‐1 family, is recognized as a key anti‐inflammatory cytokine that not only suppresses innate immune responses but also plays a significant role in regulating tumorigenesis, particularly angiogenesis. Mounting evidence indicates that IL‐37 shifts the balance between pro‐ and anti‐angiogenic factors toward anti‐angiogenic signaling by suppressing critical signaling pathways, including Signal transducer and activator of transcription 3 (STAT3), Akt/mTOR, Wnt/β‐catenin, and Notch. In breast, lung, colorectal, and hepatocellular cancers, reduced IL‐37 expression has been associated with higher tumor vessel density, more advanced disease stages, and poorer prognosis.

**Recent Findings:**

In preclinical models, overexpression or administration of IL‐37 has led to decreased vascular endothelial growth factor (VEGF) levels, inhibition of hypoxia‐inducible factor‐1α (HIF‐1α), and enhanced expression of anti‐angiogenic factors, such as thrombospondin‐1 (TSP‐1). Clinically, low IL‐37 levels may serve as a potential biomarker for predicting survival and therapeutic response.

**Conclusion:**

Despite promising findings, challenges remain, including optimizing delivery systems, managing the risk of immunosuppression, and conducting controlled clinical trials. IL‐37, predominantly produced by innate immune cells and detectable in the tumor and stromal compartments, exhibits therapeutic potential by modulating inflammatory and angiogenic signaling networks within the tumor microenvironment. A deeper understanding of IL‐37's molecular interactions with key pathways, along with the design of combination approaches, could pave the way for its practical clinical application.

## 1 Introduction

Interleukin‐37 (IL‐37), a prominent member of the interleukin‐1 family, was first identified and characterized in 2000. The IL‐37‐encoding gene is located within a gene cluster near IL‐18 and IL‐1β on human chromosome 2 and shares structural and functional homology with these genes [1]. To date, five splice variants of IL‐37 have been identified (IL‐37a–e), among which the *IL‐37b* isoform has been the most extensively studied in functional analyses [2]. Initially recognized for its anti‐inflammatory properties, IL‐37 was established as a negative regulator of innate immune responses. Evidence demonstrates that this cytokine can suppress the production of proinflammatory cytokines and attenuate inflammation across various disease models [3]. Given the absence of a murine ortholog for IL‐37, its functional studies are conducted in transgenic models expressing human IL‐37 [4]. IL‐37 expression is inducible and present at low levels under physiological conditions, but is significantly upregulated by inflammatory stimuli such as lipopolysaccharide (LPS), IL‐1β, and TNF‐α [5].

IL‐37 is produced by innate immune cells, such as monocytes, macrophages, and dendritic cells, and is also found in epithelial and tumor cells. Its expression increases after Toll‐like receptor (TLR) activation, pathogen exposure, and cytokine stimulation. Beyond acute inflammation, IL‐37 is upregulated in chronic inflammation and tumor environments, indicating it may regulate persistent immune responses [6, 7]. IL‐37 broadly suppresses innate and adaptive immunity by inhibiting NF‐κB, MAPK, and STAT3 pathways, reducing IL‐6, IL‐1β, TNF‐α, and other mediators, and modulating immune and metabolic reprogramming. These properties underpin its role in tumor progression and angiogenesis.

IL‐37 exhibits dual functionality at intracellular and extracellular levels. Intracellularly, it translocates to the nucleus and suppresses target gene transcription through interaction with Smad3. Extracellularly, it binds to the IL‐18 receptor α subunit and IL‐1R8 (also known as SIGIRR), thereby activating anti‐inflammatory signaling pathways [8]. Beyond its anti‐inflammatory roles, emerging evidence suggests that IL‐37 may exert dual functions in tumorigenesis and angiogenesis, particularly by modulating the tumor microenvironment and regulating the balance between pro‐ and anti‐angiogenic factors [9].

Angiogenesis is a critical process in tumor growth and progression, enabling tumors to overcome oxygen diffusion limitations and facilitate metastasis. This phenomenon results from a complex interplay between pro‐ and anti‐angiogenic factors, with vascular endothelial growth factor (VEGF) playing a central role [10]. The “angiogenic switch” is activated when the balance tilts in favor of pro‐angiogenic factors, leading to the sprouting of new blood vessels from pre‐existing vasculature and the formation of an unstable, leaky network of tumor‐associated vessels [11]. In addition to VEGF signaling through VEGFR2, multiple pathways, including Notch‐Dll4, PI3K/AKT, STAT3, NF‐κB, and TGF‐β signaling, coordinate endothelial proliferation, migration, and vascular remodeling in the tumor microenvironment [12, 13, 14]. Tumor‐associated macrophages, myeloid‐derived suppressor cells, hypoxia‐induced HIF‐1α activation, and cytokines such as IL‐6 and TGF‐β further shape angiogenic outcomes by dynamically modulating endothelial behavior and stromal interactions [15, 16]. These abnormal vessels contribute to hypoxia, local inflammation, and enhanced metastatic potential.

Given the complexity of this multilevel regulatory network, cytokines that modulate inflammatory signaling, such as IL‐37, may influence angiogenesis at several distinct stages, including suppression of pro‐angiogenic transcriptional programs, modulation of endothelial activation, or indirect alteration of tumor‐derived angiogenic mediators. Importantly, accumulating evidence indicates that IL‐37 does not exert a uniform anti‐angiogenic effect but rather displays context‐dependent activity, influenced by tumor type, receptor expression, and the microenvironment.

Unlike prior reviews that primarily emphasize the broad anti‐inflammatory and immunomodulatory roles of IL‐37 in cancer, this review specifically focuses on its mechanistic interplay with tumor angiogenesis. By synthesizing emerging data on IL‐37–mediated regulation of STAT3, NF‐κB, Smad3, and VEGF‐driven signaling networks, we examine how context‐dependent effects may account for inconsistencies observed across tumor types. Given that existing evidence on IL‐37 in tumor‐associated angiogenesis remains fragmented and, at times, contradictory, a structured mechanistic reassessment is warranted. Accordingly, this review critically evaluates the molecular pathways linking IL‐37 to angiogenic regulation across distinct tumor contexts, highlights tumor‐type variability and microenvironmental influences, and identifies key translational gaps that must be addressed to clarify its therapeutic potential in cancer.

## 2 Mechanistic Overview of Tumor Angiogenesis: Implications for IL‐37 Modulation

Tumor angiogenesis is initiated by the “angiogenic switch,” a process driven primarily by hypoxia‐induced stabilization of hypoxia‐inducible factor‐1α (HIF‐1α), which transcriptionally activates VEGF and other pro‐angiogenic mediators [17]. VEGF binding to VEGFR2 on endothelial cells triggers downstream signaling cascades, including PI3K/AKT, MAPK/ERK, and Signal transducer and activator of transcription 3 (STAT3) pathways, promoting endothelial proliferation, migration, and survival [18].

Following endothelial activation, coordinated Notch‐Dll4 signaling regulates tip and stalk cell differentiation, ensuring controlled sprouting and directional vessel growth [19]. Concurrently, extracellular matrix remodeling mediated by matrix metalloproteinases (MMP‐2 and MMP‐9) facilitates endothelial invasion into the surrounding stroma [20]. Subsequent vessel maturation requires recruitment of pericytes through PDGF‐B signaling and stabilization via angiopoietin–Tie2 interactions [21].

Beyond endothelial‐intrinsic mechanisms, the tumor microenvironment critically shapes angiogenesis. Tumor‐associated macrophages, myeloid‐derived suppressor cells, and inflammatory cytokines such as IL‐6 and TGF‐β amplify pro‐angiogenic signaling networks, often through activation of NF‐κB and STAT3 pathways [22, 23]. Dysregulated crosstalk among these pathways results in structurally abnormal and functionally inefficient tumor vasculature.

**FIGURE 1:**
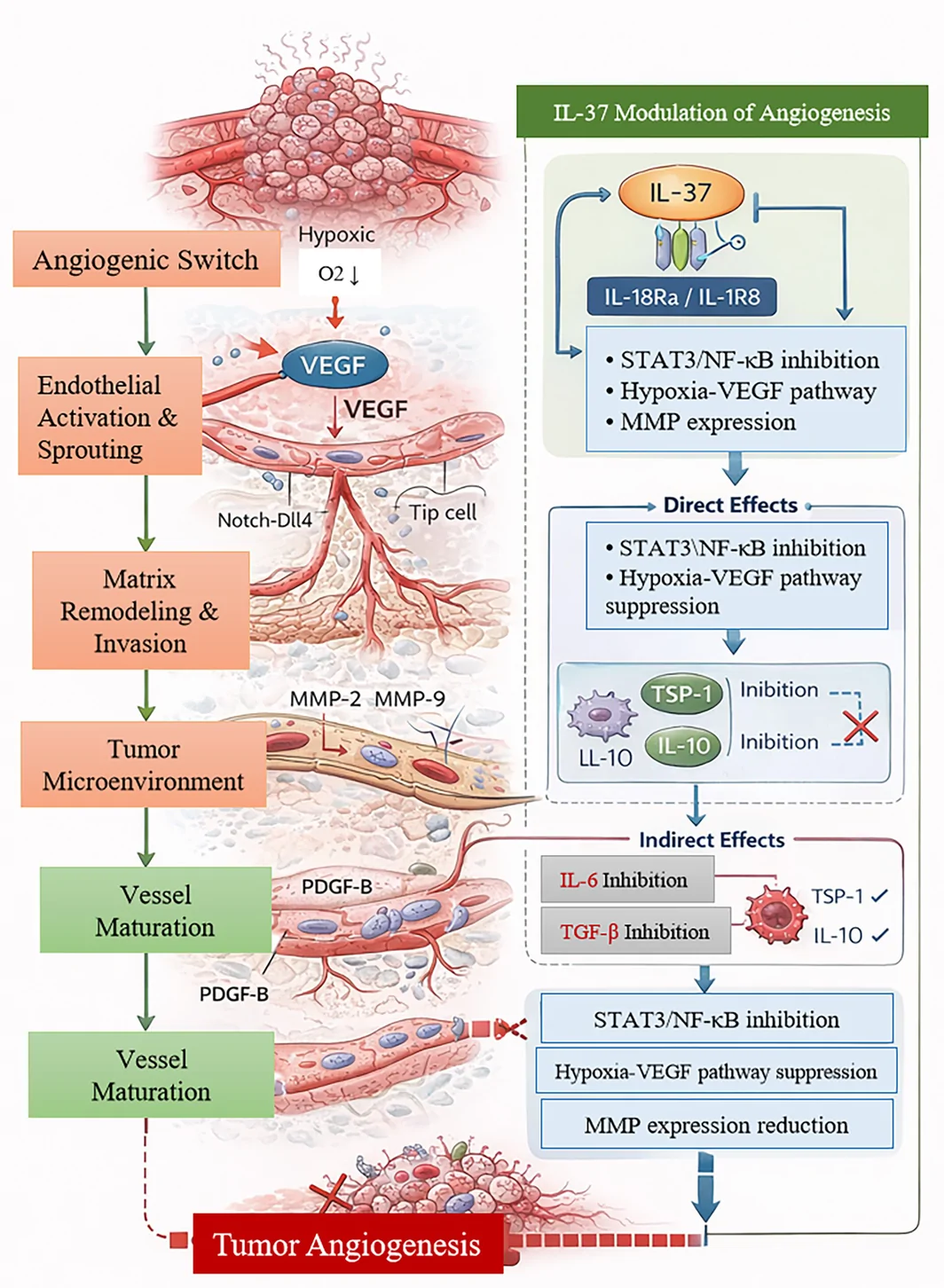
Schematic of tumor angiogenesis and IL‐37's modulation. Hypoxia triggers VEGF and endothelial sprouting via VEGFR2, Notch–Dll4, and matrix remodeling. IL‐37 binds IL‐18Rα and IL‐1R8, suppressing STAT3 and NF‐κB, reducing VEGF and MMP expression, and modulating cytokine production. These effects collectively decrease tumor angiogenesis contextually. Created this figure with BioRender.com.

Given that IL‐37 is a potent modulator of inflammatory and STAT3/NF‐κB–dependent signaling, it is mechanistically plausible that this cytokine may influence angiogenesis at multiple levels: (1) suppression of hypoxia‐driven pro‐angiogenic transcriptional programs, (2) indirect modulation of tumor‐derived VEGF and MMP expression, and (3) alteration of immune cell–mediated angiogenic support within the tumor microenvironment. Figure 1 provides a schematic overview of these interconnected mechanisms, highlighting the points at which IL‐37 interfaces with endothelial signaling pathways and tumor microenvironment–derived angiogenic mediators. Understanding these mechanistic intersections is essential for interpreting the context‐dependent vascular effects of IL‐37.

## 3 IL‐37 and Its Overall Effects on Angiogenesis

Experimental studies have provided direct evidence that IL‐37 modulates tumor‐associated angiogenesis through both indirect cytokine suppression and direct endothelial regulation. In a transgenic fibrosarcoma model, Yang et al. demonstrated that IL‐37 expression significantly reduced intratumoral microvessel density, accompanied by decreased secretion of VEGF and IL‐6 and increased expression of thrombospondin‐1 (TSP‐1), suggesting a shift toward an anti‐angiogenic molecular profile [24].

In addition to tumor‐mediated effects, IL‐37 has been shown to directly suppress endothelial cell function. Jiang et al. reported that recombinant IL‐37 inhibited proliferation, migration, and tube formation in human umbilical vein endothelial cells (HUVECs), indicating a cell‐autonomous inhibitory role in vascular formation [8, 25].

Collectively, these findings indicate that IL‐37 can attenuate angiogenesis via multiple mechanisms, including downregulation of pro‐angiogenic mediators (VEGF, IL‐6, IL‐8), suppression of STAT3‐ and NF‐κB‐dependent signaling, and induction of anti‐angiogenic factors such as TSP‐1 and IL‐10. However, emerging data from other tumor contexts suggest that these effects may not be universally inhibitory, underscoring the importance of tumor type, receptor expression, and microenvironmental composition in determining the net vascular outcome.

## 4 IL‐37 in Specific Cancer Types

### 4.1 Breast Cancer

In breast cancer, IL‐37 has been identified as a potent negative regulator of tumor angiogenesis. Wang et al. analyzed tissue samples from breast cancer patients and found that IL‐37 expression inversely correlates with microvascular density and tumor progression. In vitro and in vivo experiments demonstrated that IL‐37 suppresses tumor growth and metastasis by reducing VEGF expression and inhibiting neovascularization [26]. In another study, Li et al. investigated the molecular mechanisms underlying IL‐37's anti‐angiogenic effects in breast cancer cell models. Their results revealed that IL‐37 downregulates pro‐angiogenic factors, such as VEGF and matrix metalloproteinase‐9 (MMP‐9), by inhibiting STAT3 signaling, ultimately reducing tumor growth and angiogenesis in a breast cancer mouse model [27].

### 4.2 Lung Cancer

In lung cancer, particularly non‐small cell lung cancer (NSCLC), substantial evidence supports the anti‐angiogenic role of IL‐37. Ge et al. showed that elevated IL‐37 expression in NSCLC cells significantly reduces tumor growth and vascular density in murine models. This effect was attributed to the suppression of the Akt/mTOR signaling pathway, resulting in decreased expression of pro‐angiogenic factors, including VEGF and basic fibroblast growth factor (bFGF) [8]. Furthermore, Chen et al. explored the role of IL‐37 in hypoxia‐induced angiogenic responses. Their findings indicated that IL‐37 inhibits angiogenic activity in NSCLC cells by suppressing HIF‐1α expression and its downstream targets, such as VEGF [28].

### 4.3 Colorectal Cancer (CRC)

In CRC, growing evidence suggests that IL‐37 exerts protective, anti‐tumor, and anti‐angiogenic effects. IL‐37 expression is significantly reduced in tumor tissues, and this downregulation is associated with advanced disease stage, metastasis, and poorer prognosis. In a pivotal study by Yan et al. low IL‐37 levels were linked to higher microvessel density and increased VEGF expression in CRC tissues. The researchers demonstrated that IL‐37 suppresses tumor growth and angiogenesis by inhibiting the IL‐6/STAT3 pathway, thereby reducing cancer cell proliferation and invasion [29].

In a clinical study involving training (*n* = 337) and validation (*n* = 245) cohorts, the same group reported that low IL‐37 expression in colorectal tumors correlates with more aggressive malignant features (higher TNM stage, invasion, and metastasis). Multivariate statistical analyses confirmed IL‐37 as an independent prognostic indicator of survival. Additionally, a nomogram based on IL‐37 levels accurately predicted survival and disease progression risk [30]. At the molecular level, IL‐37 inhibits proliferation, migration, invasion, and cancer stem‐like properties by suppressing the Wnt/β‐catenin pathway and downregulating its target genes (e.g., *c‐Myc* and *Cyclin D1*). There is also evidence that IL‐37 expression enhances the sensitivity of CRC cells to chemotherapy [29].

In a broader review, low levels of IL‐37 and IL‐38 in colorectal tumors were associated with increased local inflammation, abnormal angiogenesis, and disease progression. The authors concluded that IL‐37 exerts anti‐tumor effects through multiple mechanisms, including modulation of tumor immunity, reduction of inflammation, inhibition of angiogenesis, and regulation of key signaling pathways, making it a potential therapeutic target for controlling CRC progression [31].

### 4.4 Hepatocellular Carcinoma (HCC)

IL‐37 has shown promising anti‐angiogenic effects in HCC. Li et al. investigated the role of IL‐37 in HCC progression and found that its expression negatively correlates with tumor size, vascular invasion, and poor prognosis in HCC patients. In vitro and in vivo experiments revealed that IL‐37 suppresses HCC growth and angiogenesis by inhibiting the IL‐6/STAT3 signaling pathway [32]. Another study reported significantly lower IL‐37 expression in HCC tissues compared to adjacent non‐tumor tissues, with a negative correlation with serum α‐FP levels. In HepG2 and MHCC97H cell lines, IL‐37 induction reduced NF‐κB protein levels by 56.50% and 30.52%, respectively (*p* < 0.05), highlighting its regulatory role in the NF‐κB inflammatory pathway and its association with poor prognosis in HCC [33]. Liu et al. further demonstrated that IL‐37 expression was reduced in 85.15% (86/101) of HCC samples, and this downregulation was associated with larger tumor size, vascular invasion, and worse survival. Mechanistically, induced IL‐37 shifted Smad3 phosphorylation from the oncogenic JNK/pSmad3L/*c‐Myc* isoform to the tumor‐suppressive pSmad3C/p21 isoform. These results suggest that the IL‐37–pSmad3 axis plays a critical role in HCC progression [34].

### 4.5 Other Cancer Types

Beyond breast, lung, and CRCs, the anti‐angiogenic effects of IL‐37 have been noted in other malignancies. In cervical cancer, Wu et al. reported that increased IL‐37 expression inhibits tumor growth and angiogenesis by suppressing the PI3K/Akt/mTOR signaling pathway. This inhibition was accompanied by reduced expression of VEGF and HIF‐1α, both key drivers of tumor angiogenesis [25]. In renal cell carcinoma (RCC), Jiang et al. showed that IL‐37 significantly inhibits cell migration and invasion by downregulating VEGF and MMP‐2 and MMP‐9. This study also found that high IL‐37 expression in tumor samples correlates with more prolonged patient survival, reinforcing its role as a favorable prognostic marker [24]. In melanoma, Wang et al. found that IL‐37 suppresses tumor growth and vascularization by modulating the tumor microenvironment. Mechanistically, IL‐37 reduces the secretion of inflammatory cytokines such as IL‐6 and TNF‐α and inhibits the STAT3 pathway, shifting the balance between pro‐ and anti‐angiogenic factors in favor of tumor suppression [35].

Overall, these studies provide consistent evidence that IL‐37 exerts anti‐angiogenic and anti‐tumor effects across a wide range of malignancies, establishing it as an emerging therapeutic target for inhibiting cancer progression and metastasis.

## 5 Molecular Mechanisms of IL‐37 in Cancer Angiogenesis

**FIGURE 2:**
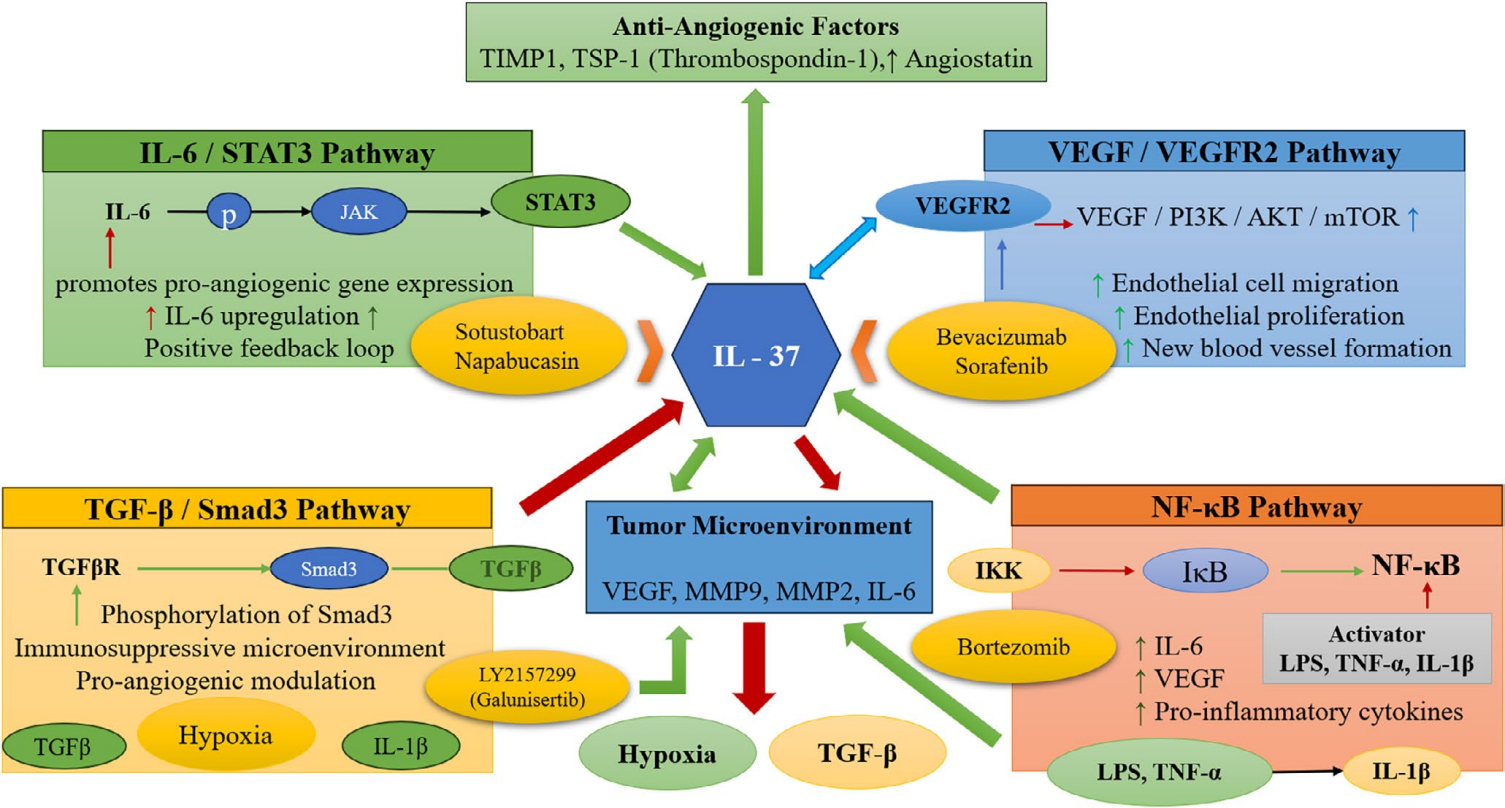
Molecular mechanisms by which IL‐37 modulates tumor angiogenesis. IL‐37 interacts with key inflammatory and angiogenic signaling pathways, including IL‐6/STAT3, VEGF/VEGFR2, TGF‐β/Smad3, and NF‐κB pathways. These pathways regulate the expression of pro‐angiogenic mediators such as VEGF, matrix metalloproteinases (MMP2 and MMP9), and interleukin‐6 (IL‐6), as well as anti‐angiogenic factors including tissue inhibitor of metalloproteinases‐1 (TIMP1), TSP‐1, and angiostatin. Pharmacologic inhibitors targeting these pathways (e.g., bevacizumab, sorafenib, galunisertib, napabucasin, bortezomib) are indicated to illustrate clinically actionable nodes within the angiogenic network. Green arrows indicate activation; red blunt arrows indicate inhibition. Created this figure with BioRender.com.

The anti‐angiogenic effects of IL‐37 are mediated through a complex network of molecular pathways and cellular regulators that influence endothelial cell growth, survival, and function, as well as the tumor microenvironment. To provide a unified mechanistic perspective, an integrated signaling model is presented in Figure 2.

### 5.1 Suppression of Pro‐Angiogenic Factors

IL‐37 inhibits the expression of key angiogenesis‐associated genes, including VEGF, IL‐6, and bFGF, thereby reducing endothelial cell stimulation and limiting neovascularization. Across multiple studies, higher IL‐37 expression has been associated with lower VEGF levels in breast, lung, and CRCs [8, 26, 36].

### 5.2 Inhibition of Angiogenic Signaling Pathways

IL‐37 targets several molecular pathways essential for angiogenesis induction. The most prominent inhibited pathways include:

- STAT3 pathway, which regulates VEGF and MMP‐9 expression [32];

Moreover, the cooperative interaction between STAT3 and HIF‐1α further amplifies angiogenic gene expression in hypoxic tumor environments [37].

- Akt/mTOR pathway, which controls endothelial cell survival and proliferation [8, 25];
- Wnt/β‐catenin pathway, critical for cell migration and invasion [38];
- Notch pathway, which governs endothelial differentiation and vascular branching [39].

Inhibition of these pathways halts endothelial cell cycle progression and prevents the formation of vascular networks.

### 5.3 Modulation of the Tumor Microenvironment

By suppressing proinflammatory cytokine production and reducing M2 macrophage infiltration, IL‐37 shifts the balance between pro‐ and anti‐angiogenic factors in the tumor microenvironment toward vascular inhibition [26, 36]. These changes alleviate hypoxia and slow tumor growth.

### 5.4 Direct Effects on Endothelial Cells

IL‐37 directly modulates endothelial cell behavior, inhibiting proliferation, migration, and tube formation [24]. These effects are likely mediated by the suppression of Akt and STAT3 phosphorylation, as well as the downregulation of adhesion molecules.

### 5.5 Regulation of Hypoxic Response

IL‐37 suppresses the hypoxia pathway by reducing HIF‐1α expression and its downstream targets, such as VEGF and GLUT‐1, thereby decreasing hypoxia‐driven angiogenesis in cancer cells [28].

Overall, IL‐37 plays a multifaceted role in inhibiting cancer‐associated angiogenesis by coordinating the suppression of multiple molecular pathways and modulating the tumor microenvironment, highlighting its significant potential for developing novel anti‐angiogenic therapeutic strategies (Figure 2).

## 6 Dual and Context‐Dependent Effects of IL‐37 on Angiogenesis

Although most preclinical studies have highlighted IL‐37's anti‐angiogenic properties, emerging evidence suggests that its role in vascular regulation may be context‐dependent rather than universally inhibitory. IL‐37 is primarily recognized as an anti‐inflammatory cytokine that suppresses excessive immune activation through interaction with IL‐18Rα and SIGIRR (IL‐1R8), leading to downregulation of NF‐κB, STAT3, and MAPK signaling pathways [40, 41]. In tumor settings characterized by inflammation‐driven angiogenesis, this suppressive effect often results in reduced VEGF production and impaired endothelial cell proliferation.

However, IL‐37's anti‐inflammatory activity may produce indirect vascular effects that vary across biological contexts [42]. In certain inflammatory or tissue repair environments, suppression of excessive immune activation may stabilize endothelial integrity and promote vascular normalization rather than regression. Moreover, tumor heterogeneity, including differences in dominant oncogenic pathways, cytokine milieu, and receptor expression profiles, may influence whether IL‐37 signaling ultimately suppresses or modulates angiogenesis [25]. For example, tumors driven predominantly by STAT3‐mediated VEGF overexpression may be more sensitive to IL‐37–mediated inhibition, whereas tumors relying on alternative pro‐angiogenic pathways may show attenuated responses.

These discrepancies highlight the need for caution in interpreting IL‐37 as a uniformly anti‐angiogenic molecule. Instead, its vascular effects appear to depend on tumor type, microenvironmental inflammation, cellular source of IL‐37, and relative dominance of downstream signaling networks. A systematic and comparative evaluation across experimental models is therefore necessary before generalizable conclusions can be drawn.

These findings underscore the need to interpret IL‐37–mediated angiogenic modulation within a defined biological context rather than extrapolate across tumor systems. Table 1 provides a structured comparison of pro‐ and anti‐angiogenic outcomes across experimental models to facilitate a balanced and critical evaluation.

**TABLE 1:** Context‐dependent effects of IL‐37 on angiogenesis across experimental models and tumor types.

Model	Cancer/Condition	Experimental system	Effect on angiogenesis	Dominant pathway/Mechanism	References
Murine orthotopic tumor	HCC	In vivo murine HCC model; HUVEC migration and tubule assay	Anti‐angiogenic in TME, despite direct pro‐angiogenic effects on ECs	Modulation of tumor cell pro/anti‐angiogenic factor expression; reduces MMP2, VEGF	[25]
Retinal and Matrigel angiogenesis	Developmental and pathologic angiogenesis model	Aortic ring, HUVEC proliferation, retinal neovascularization	Pro‐angiogenic (enhanced EC proliferation, capillary/tube formation)	Activation of ERK1/2 and AKT signaling in ECs	[43]
Pathological angiogenesis (w/TGF‐β)	Pathologic angiogenesis and oxygen‐induced retinopathy	HUVEC tube formation, Matrigel plug assay, mouse OIR	Pro‐angiogenic via TGF‐β signaling	IL‐37 + TGF‐β → signals through ALK1 receptor to promote angiogenesis	[42]
NSCLC	NSCLC xenograft tumors	Nude mice tumor model; HUVEC tube formation and VEGF measurement	Anti‐angiogenic in vivo (reduced MVD, suppressed VEGF)	Indirect suppression of VEGF and EC growth in the tumor context	[8]
Multiple myeloma clinical correlation	Multiple myeloma patients	Serum IL‐37 & VEGF/Ang‐2 correlation; HUVEC tube assay	Anti‐angiogenic correlation (IL‐37 inversely related to pro‐angiogenic factors)	Negative correlation with VEGF and Ang‐2 levels; inhibited tube formation	[44]

## 7 Determinants of Context‐Dependent IL‐37 Activity in Tumor Angiogenesis

The apparent contradictions in the literature regarding IL‐37 and angiogenesis may not reflect experimental inconsistency but rather biological heterogeneity [25, 41]. Several context‐defining variables likely determine whether IL‐37 exerts net pro‐angiogenic, anti‐angiogenic, or modulatory effects.

First, tumor type–specific signaling dominance appears to be a major determinant. Tumors characterized by inflammation‐driven STAT3 or NF‐κB activation, such as HCC and certain lung cancers, may be particularly sensitive to IL‐37–mediated suppression of pro‐angiogenic cytokines [8, 45]. In contrast, tumors in which angiogenesis is driven predominantly by hypoxia‐inducible pathways independent of inflammatory signaling may show attenuated responsiveness.

Second, the tumor microenvironment critically influences IL‐37 activity. The local cytokine milieu, including TGF‐β, IL‐6, VEGF, and chemokines, as well as immune cell composition (e.g., macrophage polarization states and myeloid‐derived suppressor cells), may modify IL‐37 signaling outcomes. For example, TGF‐β–rich environments have been reported to potentiate endothelial responses to IL‐37, suggesting that combinatorial signaling may shift the angiogenic balance [25, 42].

Third, the cellular source and mode of IL‐37 delivery are likely to contribute to divergent observations. Tumor cell–derived IL‐37, immune cell–derived IL‐37, and exogenous recombinant IL‐37 may engage distinct receptor dynamics and downstream pathways. Differences in IL‐18Rα and IL‐1R8 expression levels across tumor types may further modulate signaling strength and biological outcome [4, 41].

Taken together, these factors underscore that IL‐37 should not be interpreted as a universally anti‐angiogenic molecule. Instead, its function appears to be shaped by tumor‐specific signaling architecture, microenvironmental context, and cellular origin, necessitating cautious and stratified interpretation of existing data [6, 45]. Table 2 summarizes the angiogenic effects of IL‐37 in cancers.

**TABLE 2:** Summary of experimental studies evaluating the angiogenic effects of IL‐37 across cancer types.

Cancer type	Study type	Experimental model/Cell line	Intervention	Anti‐angiogenic findings	Pro‐angiogenic/Context‐dependent findings	Key pathways involved	References
Breast cancer	In vivo + in vitro	4T1 murine model; 4T1 cells	IL‐37b gene transfer (plasmid overexpression)	↓ Microvessel density; ↓VEGF; ↓MMP‐9; reduced tumor growth and metastasis	No pro‐angiogenic effect reported in this model	STAT3 inhibition	[26]
NSCLC	In vivo + in vitro	A549, H1299; xenograft mice	IL‐37 overexpression	↓VEGF; ↓bFGF; suppressed tumor angiogenesis; improved survival	No pro‐angiogenic effect reported	Akt/mTOR pathway inhibition	[8]
CRC	In vivo + clinical samples	IL‐37 transgenic mice; human CRC tissues	Endogenous IL‐37 expression analysis	↓Microvessel density; ↓IL‐6/STAT3; ↓Wnt/β‐catenin; reduced progression	Context may depend on tumor inflammatory profile; no direct pro‐angiogenic activity shown	IL‐6/STAT3; Wnt/β‐catenin	[29]
HCC	Clinical correlation + in vitro	Human HCC tissues; HCC cell lines	Expression analysis; recombinant IL‐37	↓VEGF; ↓IL‐6; ↓HIF‐1α; reduced proliferation	Low IL‐37 expression correlated with higher angiogenic markers (inverse association)	VEGF/HIF‐1α axis	[33]
Cervical cancer	In vitro	HeLa, SiHa cells	Recombinant IL‐37	↓Proliferation; ↓invasion; ↓angiogenic mediators	No pro‐angiogenic activity observed	STAT3; PI3K/Akt	[35]

*Note:* The table distinguishes anti‐angiogenic findings from context‐dependent observations and specifies experimental models and signaling pathways involved.

## 8 Therapeutic Potential and Clinical Implications

The anti‐angiogenic and immunomodulatory properties of IL‐37 position this cytokine as a promising candidate for targeted therapeutic interventions in cancer. Growing evidence indicates that IL‐37 not only inhibits tumor growth and angiogenesis but also enhances anti‐tumor immune responses. Potential therapeutic strategies in this domain include the following:

### 8.1 Recombinant IL‐37

Administration in preclinical models has demonstrated its ability to suppress tumor growth, inflammation, and neovascularization. For instance, Yang et al. reported that recombinant IL‐37 treatment in a murine fibrosarcoma model significantly reduced VEGF and IL‐6 expression while increasing TSP‐1 levels, thereby inhibiting tumor angiogenesis [36].

### 8.2 Gene Therapy

Overexpression of IL‐37 via gene therapy represents an emerging approach to induce its anti‐tumor effects. Ge et al. reported that IL‐37 overexpression in lung cancer cells led to reduced tumor growth and vascular density in in vivo models [8]. Such strategies could lead to the development of targeted viral or non‐viral vectors for IL‐37 gene delivery to tumor tissues.

### 8.3 Combination Therapy

Given IL‐37's anti‐inflammatory and immunomodulatory properties, its combination with standard cancer therapies such as chemotherapy or anti‐angiogenic agents may yield synergistic effects. Moreover, recent findings suggest that IL‐37 can serve as an adjuvant immunomodulator alongside immune checkpoint inhibitors (e.g., anti‐PD‐1 or anti‐CTLA‐4 antibodies), enhancing immunotherapy efficacy by reducing tumor immune resistance [46].

### 8.4 Biomarker Potential

IL‐37 expression levels in serum or tumor tissues may serve as diagnostic or prognostic biomarkers. Multiple studies have shown that reduced IL‐37 expression correlates with tumor progression, increased angiogenesis, and unfavorable prognosis in cancers such as HCC and breast cancer [26, 32]. Evidence suggests that IL‐37 is a versatile therapeutic molecule that modulates inflammatory, immune, and angiogenic pathways across different cancers. The experimental evidence supporting the pro‐ and anti‐angiogenic roles of IL‐37 across different malignancies is summarized in Table 3, with a clear distinction between in vitro and in vivo models, the cellular systems used, and the mechanistic pathways involved.

**TABLE 3:** Selected clinical trials targeting angiogenic and inflammatory signaling pathways mechanistically intersect with IL‐37 activity.

Drug/Intervention	Tumor type	Outcome	Pathway related to IL‐37	References
Bevacizumab (anti‐VEGF)	Metastatic colorectal cancer	Improved progression‐free survival	VEGF pathway (downstream of inflammatory signaling modulated by IL‐37)	[47]
Atezolizumab + Bevacizumab	HCC	Improved overall survival versus sorafenib	VEGF–STAT3 axis modulation	[48]
Siltuximab (anti‐IL‐6)	Advanced solid tumors	Modulation of inflammatory signaling	IL‐6/STAT3 pathway	[49]
Galunisertib (TGF‐βR1 inhibitor)	HCC	Disease stabilization in the subset	TGF‐β signaling	[50]
Bortezomib (NF‐κB pathway modulation)	Multiple myeloma	Improved survival in combination regimens	NF‐κB signaling	[44]

*Note:* Although IL‐37 itself has not yet been evaluated in oncology clinical trials, the drugability of its downstream signaling networks (VEGF, STAT3, TGF‐β, NF‐κB) highlights potential translational relevance.

The potential prognostic relevance of IL‐37 in cancer remains incompletely defined. While several studies have reported inverse correlations between IL‐37 expression and tumor stage, angiogenic markers, or overall survival, most available data are derived from retrospective cohort analyses with limited multivariate adjustment. Consequently, it remains unclear whether IL‐37 represents an independent prognostic factor or whether its predictive value is largely context‐dependent, reflecting broader inflammatory or immune landscape features within specific tumor types. Prospective studies incorporating standardized quantification methods and multivariate modeling will be necessary to determine whether IL‐37 provides independent prognostic information beyond established clinicopathological parameters.

## 9 From Preclinical Promise to Clinical Translation

Preclinical studies consistently demonstrate that IL‐37 overexpression or recombinant administration can suppress angiogenic mediators, including VEGF, IL‐6, and MMPs, across several tumor models. However, these findings largely derive from controlled experimental systems employing gene transfer techniques, transgenic mice, or high‐dose exposure to recombinant protein. Such approaches do not necessarily reflect clinically achievable pharmacologic conditions [51].

Translational progression of IL‐37–based strategies faces several unresolved challenges. First, optimal dosing parameters remain undefined, particularly given the absence of standardized comparisons between endogenous physiological levels and experimentally induced overexpression [52]. Second, the route of administration, whether systemic recombinant protein delivery, localized gene transfer, or nanoparticle‐based targeting, requires careful evaluation in terms of bioavailability and tissue specificity. Third, pharmacokinetic and stability profiles of IL‐37 have not yet been systematically characterized in oncologic settings [53]. Finally, patient stratification may prove critical, as tumors with high baseline STAT3/NF‐κB activation or specific receptor expression patterns (IL‐18Rα/IL‐1R8) may respond differently to IL‐37 modulation.

Collectively, these considerations highlight that while IL‐37 demonstrates compelling biological promise, substantial work remains before clinical viability can be realistically established.

## 10 Translational Context of IL‐37‐Related Angiogenic Pathways

Although IL‐37–based therapeutic strategies have not yet advanced to clinical testing in oncology, several angiogenic and inflammatory pathways that mechanistically intersect with IL‐37 signaling are well established as clinically druggable targets. IL‐37 has been shown to modulate VEGF expression, inhibit STAT3 and NF‐κB activation, and influence TGF‐β–mediated signaling cascades that play central roles in tumor angiogenesis and progression.

Anti‐VEGF strategies, such as bevacizumab and VEGF‐targeted combination regimens, have demonstrated significant survival benefits across multiple malignancies, including CRC and HCC [47, 48]. Similarly, therapeutic inhibition of IL‐6/STAT3 signaling has been explored in advanced solid tumors and inflammatory malignancies, supporting the clinical relevance of inflammatory‐angiogenic crosstalk [49]. Targeting the TGF‐β pathway has also shown clinical activity in selected cancer subsets, particularly in tumors characterized by a pro‐fibrotic and immunosuppressive microenvironment [50]. Furthermore, modulation of NF‐κB–associated signaling remains an established strategy in hematologic malignancies and inflammation‐driven cancers [44].

Representative clinical trials targeting these IL‐37–related pathways are summarized in Table 3. While these studies do not directly evaluate IL‐37 as a therapeutic agent, they collectively demonstrate that the angiogenic and inflammatory networks influenced by IL‐37 are clinically actionable. This translational landscape supports the biological plausibility of IL‐37 as a future immunomodulatory strategy, while underscoring the need for rigorous preclinical optimization before clinical implementation.

While the majority of experimental studies report anti‐angiogenic effects of IL‐37, not all findings are uniformly suppressive. In certain tumor contexts, IL‐37 expression has shown neutral associations with angiogenic markers or indirect correlations that may reflect broader inflammatory regulation rather than direct vascular inhibition. For example, clinical correlation studies in HCC have demonstrated inverse associations between IL‐37 levels and pro‐angiogenic mediators without establishing direct causality. Moreover, variations in receptor expression, baseline inflammatory signaling intensity, and tumor microenvironment composition may attenuate or modify IL‐37 responsiveness. These observations reinforce the concept that IL‐37 functions as a context‐dependent immunomodulator rather than a universally anti‐angiogenic factor, underscoring the need for tumor‐specific mechanistic evaluation.

## 11 Challenges and Future Directions

Although recent studies have elucidated the protective and anti‐angiogenic roles of IL‐37 across various cancers, translating these findings from the laboratory to the clinic remains fraught with significant challenges. These obstacles and future directions can be summarized across several key domains:

### 11.1 Optimization of Delivery Systems

A primary barrier is designing efficient systems for the stable, targeted delivery of IL‐37 to tumor tissues. Safety risks and host immune responses limit the use of viral vectors, whereas emerging nanoparticle, hydrogel, and lipid‐based platforms offer promising alternatives. Future investigations should focus on controlled, tumor‐specific delivery [51, 52].

### 11.2 Safety and Side Effects

Given IL‐37's broad anti‐inflammatory effects, there is a risk of systemic immunosuppression or disruption of natural immune defenses. Thorough safety assessments in long‐term animal models and early‐phase clinical trials are essential to establish safe and sustainable dosing [54].

### 11.3 Tumor Resistance Mechanisms

Similar to other anti‐angiogenic therapies, tumors may develop resistance to IL‐37 through activation of alternative pathways (e.g., FGF or Notch) or metabolic reprogramming. Future studies must identify these adaptive mechanisms and develop countermeasures [55].

### 11.4 Combination Therapies

IL‐37 holds potential as a versatile agent in combination regimens with chemotherapy, immunotherapy, or anti‐angiogenic drugs. Preliminary data suggest synergistic effects with immune checkpoint inhibitors. Further research is needed to determine optimal combinations, dosages, and treatment sequences.

### 11.5 Clinical Trials

Despite promising preclinical evidence, controlled clinical trials evaluating the efficacy and safety of IL‐37‐based therapies in cancer patients remain scarce. Although several downstream angiogenic and inflammatory pathways modulated by IL‐37 have been successfully targeted in clinical oncology (see Table 3), IL‐37 itself has not yet progressed to early‐phase clinical testing. Conducting well‐designed translational studies is therefore a critical step in validating its therapeutic potential.

### 11.6 Deeper Understanding of Molecular Mechanisms

Future studies should precisely identify IL‐37's downstream targets, its interactions with key signaling pathways (STAT3, NF‐κB, HIF‐1α, Notch), and its crosstalk with immune and endothelial cells within the tumor microenvironment. This deeper insight could serve as the foundation for developing novel interleukin‐based therapeutics.

The current body of evidence evaluating IL‐37 in tumor angiogenesis is characterized by substantial heterogeneity across tumor types, experimental systems, and biological contexts. Differences in tumor origin, inflammatory landscape, receptor expression profiles (IL‐18Rα and IL‐1R8), and baseline activation of signaling pathways such as STAT3 and NF‐κB may critically influence IL‐37 responsiveness. In addition, variability in experimental design, including transient overexpression systems, stable gene transfer models, recombinant protein administration, xenograft assays, and human IL‐37 transgenic mouse models, may account for divergent findings reported in the literature. Notably, gene overexpression approaches may amplify anti‐angiogenic effects beyond those observed in patient‐derived tissues, whereas the absence of a murine ortholog necessitates reliance on transgenic systems that may not fully recapitulate endogenous regulatory dynamics.

Consequently, current evidence does not yet support universal generalizations regarding the angiogenic function of IL‐37. Instead, accumulating data points toward a context‐dependent regulatory role, shaped by tumor‐specific inflammatory tone, microenvironmental composition, and pathway dominance. However, rather than limiting the relevance of IL‐37 as a therapeutic candidate, this variability underscores the need for a context‐stratified, mechanistic synthesis. By integrating findings across diverse models and malignancies, this review highlights recurring signaling intersections and delineates conditions under which IL‐37 exerts anti‐angiogenic or context‐modulated effects. Future investigations employing standardized experimental frameworks, comparative tumor models, and physiologically relevant dosing strategies will be essential to resolve inconsistencies and establish more definitive translational conclusions.

In summary, despite technical and biological challenges, IL‐37 remains an emerging focal point in anti‐angiogenic and immunomodulatory cancer therapies. Advances in targeted delivery, safety evaluation, and combination strategies could pave the way for successful clinical application of this molecule.

## 12 Conclusion

IL‐37, as a multifaceted cytokine, plays a pivotal role in suppressing tumor growth and angiogenesis. By inhibiting pro‐angiogenic factors such as VEGF and HIF‐1α and targeting key signaling pathways, including STAT3, Akt/mTOR, Wnt/β‐catenin, and Notch, IL‐37 shifts the tumor microenvironment toward an anti‐angiogenic and immunoregulated state. Experimental evidence demonstrates that elevated IL‐37 expression reduces tumor proliferation, invasion, and metastasis, while clinical findings highlight its potential as a biomarker and prognostic indicator across various cancers.

Although preclinical findings suggest that IL‐37 may function as a negative regulator of tumor angiogenesis, several IL‐37–specific challenges must be addressed before therapeutic translation. First, accumulating evidence indicates context‐dependent dual activity, whereby IL‐37 may exert anti‐angiogenic effects in certain tumor settings while indirectly supporting vascular remodeling under distinct inflammatory or hypoxic conditions. Second, marked tumor‐type variability has been observed, with differential receptor expression (IL‐18Rα and IL‐1R8), downstream pathway engagement (STAT3, NF‐κB, Smad3), and baseline inflammatory tone influencing functional outcomes. Third, standardized dosing strategies remain undefined, particularly given differences between endogenous expression, gene‐transfer models, and recombinant protein administration. Finally, given IL‐37's broad immunoregulatory capacity, potential off‐target effects in normal tissues, particularly on vascular homeostasis and systemic immune balance, warrant careful evaluation.

## Author Contributions

**Elahe Saberi Teimourian:** validation, investigation, writing – original draft. **Mohammad Ali Karimi:** investigation, writing – original draft, validation. **Edessa Negera:** data curation. **Ghazaleh Behrouzian Fard:** formal analysis. **Anas Sattar:** software. **Mahdi Hosseini Bafghi:** project administration, supervision, writing – review and editing, validation. **Nirusha Weerasinghe:** writing – review and editing, validation, supervision, project administration.

## Funding

The authors have nothing to report.

## Ethics Statement

The authors have nothing to report.

## Consent

The authors have nothing to report.

## Conflicts of Interest

The authors declare no conflicts of interest.

## Data Availability

The data that support the findings of this study are available from the corresponding author upon reasonable request.
